# Perioperative immune checkpoint inhibition for colorectal cancer: recent advances and future directions

**DOI:** 10.3389/fimmu.2023.1269341

**Published:** 2023-11-13

**Authors:** Jiao-Ting Chen, Yu-Wen Zhou, Ting-Rui Han, Jun-Lun Wei, Meng Qiu

**Affiliations:** ^1^ Department of Colorectal Cancer Center, West China Hospital, Sichuan University, Chengdu, China; ^2^ West China School of Medicine, Sichuan University, Chengdu, China

**Keywords:** colorectal cancer, perioperative therapy, immune checkpoint inhibition, microsatellite instability-high, mismatch repair deficiency, mismatch repair proficiency, microsatellite stable

## Abstract

For colorectal cancer (CRC), surgical resection remains essential for achieving good prognoses. Unfortunately, numerous patients with locally advanced CRC and metastatic CRC failed to meet surgical indications or achieve pathological complete response after surgery. Perioperative therapy has been proven to effectively lower tumor staging and reduce recurrence and metastasis. Immune checkpoint inhibitors (ICIs) have shown unprecedented prolongation of survival time and satisfactory safety in patients with high microsatellite instability/deficient mismatch repair (MSI-H/dMMR), while the therapeutic effect obtained by patients with mismatch repair-proficient or microsatellite stable (pMMR/MSS) was considered minimal. However, recent studies found that certain CRC patients with dMMR/MSI-H presented intrinsic or acquired immune resistance, and pMMR/MSS CRC patients can also achieve better efficacy. Therefore, more predictors are required for screening patients with potential clinical benefits. Since the discovery of synergistic effects between immunotherapy, chemotherapy, and radiotherapy, different immunotherapy-based therapies have been applied to the perioperative therapy of CRC in an increasing number of research. This review comprehensively summarized the past and current progress of different combinations of immunotherapy in perioperative clinical trials for CRC, focusing on the efficacy and safety, and points out the direction for future development.

## 1 Introduction

Colorectal cancer (CRC) is the third most common cancer and the leading cause of cancer death worldwide (1–4). Due to the lack of early symptoms, 36% of patients were diagnosed with locally advanced CRC (LACRC) (stage II (cT3–4, N0)/stage III (any cT, N+)), and 22% presented with distant metastasis (5). The perioperative therapy (days before and after surgery) is of great significance in promoting tumor downgrading and reducing the local recurrence and metastasis, including neoadjuvant (preoperative) therapy and adjuvant (postoperative) therapy (6–9). Given the compelling long-term durable remission in metastatic CRC (mCRC), immune checkpoint inhibitors (ICIs) have attracted great attention in the perioperative therapy of CRC. DNA mismatch repair (MMR) and Microsatellite instability (MSI) are considered important predictors of sensitivity for immunotherapy-based strategies (10) DNA mismatch repair (MMR) is an important pathway to maintain genomic stability (10, 11). Microsatellites are highly polymorphic repetitive DNA sequences in the human genome and MSI is defined as genomic instability in cancer cells due to a deficiency in MMR (dMMR) (10–12). MSI CRC accounts for 15% of all sporadic CRC, which can be divided into MSI-high (MSI-H) and MSI-low (MSI-L) according to the frequency of microsatellite marker instability (10, 11, 13). The remaining CRC is classified as microsatellite stable (MSS), with proficiency in MMR (pMMR) (10, 11). dMMR/MSI-H CRC is associated with a higher tumor mutation burden and neoantigen load and more lymphocyte infiltration than pMMR/MSS/MSI-L CRC (10, 11, 14).

dMMR/MSI-H CRC patients, whose sensitivity to ICIs is significantly higher than that of patients with pMMR/MSS/MSI-L, have derived notable pathological responses from neoadjuvant immunotherapy (14–17). However, 40% -60% of MSI-H CRC are inherently resistant to immunotherapy (14, 18) Therefore, the main challenge is to provide more benefits of immunotherapy for the majority of patients with pMMR, MSS, MSI-L, or insensitive MSI-H CRC (19) Fortunately, it is discovered that there is a synergistic effect between immunotherapy, chemotherapy, and radiotherapy (20, 21). An increasing number of clinical trials have explored the efficacy and safety of different immunotherapy-based therapies in the perioperative period (6, 17). Therefore, this article comprehensively reviewed previous achievements and the latest progress of different immunotherapy combination therapies in the perioperative period, which may provide new therapy strategies for CRC patients to achieve better efficacy and safety, as well as the mechanism of immunotherapy combination therapy and promising predictors to identify the patients with potential benefits.

## 2 Overview of immunotherapy for colorectal cancer

### 2.1 Current status of immunotherapy for colorectal cancer

In 2015, after a phase 2 clinical trial first proved that MSI CRC was a potential beneficiary, ICIs has been explored more extensively in CRC (22). Thereafter the impressive efficacy and safety of CheckMate-142 (23) and KEYNOTE-177 (16) in the treatment of dMMR/MSI-H mCRC promoted the Food and Drug Administration (FDA) ‘s approval of pembrolizumab, a programmed cell death-1 (PD-1) inhibitor, as the first-line treatment for MSI-H advanced CRC. Recently, ipilimumab combined with nivolumab, inhibitors of a cytotoxic T-lymphocyte-associated protein 4 (CTLA-4) and PD-1, has also been granted approval by the FDA for the treatment of dMMR/MSI-H mCRC (15, 24). The encouraging results motivated researchers to investigate the application of immunotherapy in the perioperative period of CRC. Recently, studies on immunotherapy combined with chemoradiotherapy and targeted drugs have been emerging. Additionally, various immunotherapy strategies have been developed to change the situation of “cold tumor” treatment, such as oncolytic virus (25, 26), cytokine therapy (27), and chimeric antigen receptor T cell therapy (28, 29).

At present, the MMR/MSI system has become the most important classification standard for CRC (30, 31). dMMR/MSI-H represents a good prognosis in early-stage CRC, while in metastatic disease it seems to confer a poor prognosis (10). Moreover, there is evidence showing that patients with dMMR/MSI-H CRC can obtain high reactivity of ICIs therapy, but for a majority of patients with pMMR or MSS, the clinical benefits from ICIs are generally minimal (12, 14, 17, 18, 32–37). However, recent studies have shown that certain CRC patients with MSI-H presented intrinsic or acquired immune resistance, while the patients with pMMR/MSS can also achieve a higher pathological complete response (pCR) rate (38, 39). Therefore, the optimal biomarkers for screening patients with potential clinical benefits are needed (40).

### 2.2 Potential predictive indicators for perioperative immunotherapy

Currently, an increased tumor mutation burden has been observed in MSI-H CRC patients who benefit from immunotherapy, as well as MSS CRC patients, which is considered another effective biomarker (14, 19, 32). Moreover, research has identified two distinct subtypes, MSI-H1 and MSI-H2, each with different prognostic implications (41). Notably, the MSI-H1 subgroup, enriched with M2 macrophages and characterized by high PD-L2 expression, tends to indicate a lower survival rate (41). Among dMMR CRCs, beta-2-microglobulin mutations that result in complete beta-2-microglobulin loss are associated with reduced recurrence and metastasis (19).

Assessing the extent of infiltration and co-expression of CD8^+^ and PD-1 of T cells in tumors may be warranted to predict the overall survival rate and pCR rate of pMMR patients (38, 42). Additionally, polymerase epsilon exonuclease domain mutations (POLE EDM) (43–45), guanylate binding protein 2 expression (46), and soluble PD-L1 level may also be promising indicators to identify the pMMR patients with a favorable response to ICIs. Furthermore, CMTM6 expression in M2 macrophages (47), circulating L-arginine (48), the human gastrointestinal microbiome (49), fibroblast growth factor receptor 1-3 deficiency (50), and circulating tumor DNA (45, 51) may also play crucial roles in monitoring immunotherapy efficacy.

### 2.3 The mechanism of immunotherapy combination therapy

#### 2.3.1 Immunotherapy combinations

**Figure 1:**
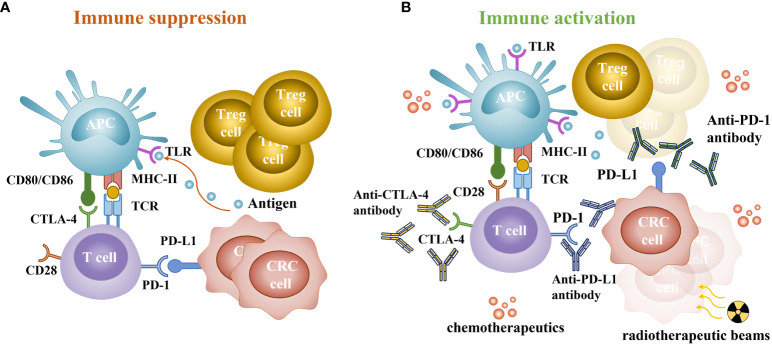
Immune status of patients with colorectal cancer. **(A)** The main way of immune suppression. CTLA-4 can competitively bind to CD80 or CD86 and inhibit activation. PD-1 is a key checkpoint for T cells, interacting with abnormally upregulated PD-L1 on cancer cells and immune cells, leading to T cell depletion and immune evasion. **(B)** The synergistic effect of chemoradiotherapy and immunotherapy. PD-1/PD-L1 and CTLA-4 checkpoint inhibitors can inhibit the negative feedback regulation of cancer cells and restore the anticancer function of T cells. Chemotherapeutics can induce immunogenic cell death and disrupt tumor escape strategies, increase the activity and quantity of toll-like receptors (TLR), promote DCs activation, deplete Treg cells, and reduce inhibition of T cells. Radiation induces tumor cell damage, releases a large number of damage-associated molecular patterns, and increases the formation and memory response of tumor-infiltrating lymphocytes.

PD-1 and CTLA-4 are key immune checkpoints for T cells, PD-1 and programmed death-ligand 1 (PD-L1) play a role by inhibiting the proximal signaling of T cell antigen receptor, while CTLA-4 weakens costimulatory signals through the co-receptor CD28, suppressing T cell activation (52, 53). Excessive activation or expression of immune checkpoints in cancer may promote malignant proliferation and metastasis (53). Therefore, PD-1-CTLA-4 inhibitors may have a synergistic effect by simultaneously inhibiting both pathways, achieving better therapeutic effects than ICIs monotherapy. However, the effective response to PD-1 blocking requires more tumor-infiltrating lymphocytes in the tumor microenvironment, which also indicates that pMMR tumors have limited efficacy owing to the lack of tumor-infiltrating lymphocytes (54) (**Figure 1**).

#### 2.3.2 Immunotherapy in combination with chemotherapy or radiotherapy

The positive effects of standard chemotherapy on tumor immunity are mainly reflected in inducing immunogenic cell death and disrupting tumor escape strategies (21). Taxanes can elevate the activity of toll-like receptors and promote the activation of dendritic cells (21). Cyclophosphamide can deplete Treg cells, reducing the inhibition of tumor-infiltrating T cells (55). Therefore, the immune enhancement effect of chemotherapy may have a synergistic effect with immunotherapy. However, owing to its non-targeted effect, excessive chemotherapy can also lead to the depletion or dysfunction of immune cells (21).

Radiation induces tumor cell damage that releases a large amount of damage-associated molecular patterns, increasing the formation of tumor-infiltrating lymphocytes and memory response (56). CD8^+^T cells release γ-interferon that upregulates the expression of PD-L1 in tumor cells, thereby exerting a synergistic effect with ICIs (20). In the previous research, when CTLA-4 inhibitor was added to radiation, radiosensitizing anti-CTLA-4 immunotherapy was observed in breast and CRC (20).

#### 2.3.3 Tumor resection and immunotherapy

Compared with adjuvant therapy, neoadjuvant ICIs can induce stronger and more extensive tumor-specific T cell responses, reduce the incidence of toxicity, have better compliance, and may even achieve clinical complete remission and avoid unnecessary surgery (42). Although Jiahao Zhu et al. believed that surgery can cause a decrease in tumor antigens, and damage to blood vessels and lymph nodes in the surgical area, leading to reduced survival benefits of adjuvant ICIs (33), effective adjuvant therapy may be necessary for diminishing small residual lesions and preventing recurrence and metastasis, especially for the patients that didn’t achieve pCR after surgery (57).

## 3 Perioperative immune checkpoint inhibition for colorectal cancer

### 3.1 ICIs monotherapy

The initial case report showed that two dMMR locally advanced rectal cancer (LARC) patients received PD-1 inhibitors (nivolumab) monotherapy to avoid adverse events (AEs) of chemoradiotherapy, which also enabled them to achieve pCR and clinical complete remission, and the latter adopted the observation and waiting (W&W) strategy without surgery (58). Moreover, the dMMR LACRC patients who are not eligible for chemotherapy, receiving anti-PD-1 inhibitors (pembrolizumab) monotherapy can also get pCR (59).

Further researches have also confirmed the value of ICIs in neoadjuvant therapy for CRC (**Table 1**). In a prospective phase 2 study (NCT04165772) (64), 12 patients with LARC received neoadjuvant PD-1 inhibitors (dostarlimab) monotherapy. Surprisingly, all patients achieved clinical complete remission, and no grade 3/4 AEs were reported. Moreover, Han, Kai, et al. (76) observed a high incidence rate (27.6%) of dMMR in 268 T4bM0 CRC patients. The pCR rate of the neoadjuvant ICIs monotherapy (pembrolizumab or nivolumab) group was significantly higher than that of the chemoradiotherapy group (70.0% *vs*. 0%). Compared with neoadjuvant chemotherapy and chemoradiotherapy, it significantly reduced the incidence of open surgery and had better disease-free survival and relatively longer overall survival. These results are also consistent with other researches (71, 72, 77, 78). Other neoadjuvant PD-1 inhibitors (toripalimab (45), sintilimab (73)) monotherapy may also have similar efficacy. A multicenter phase II study (NCT05662527) will further evaluate the efficacy and safety of neoadjuvant pembrolizumab in patients with stage I–III dMMR colon cancer (79).

**Table 1:** Clinical trials involving perioperative immunotherapy in CRC.

Types	Identifier	Trial phase	Period	Treatment	Case	PCR (case, %)	MPR (case, %)	TRAEs ≥grade 3 (case, %)	R0 resection (case, %)	Study time	References
LARC	NCT04911517	2	Neoadjuvant	CRT + concurrent Tislelizumab	50	(13, 50)	(21, 80.7)	(2, 7.7)	(27, 100)	2021/6-2024/12	(39)
LARC	NCT04518280	NA	Neoadjuvant	SCRT→ CAPOX + Toripalimab: 65 CAPOX + Toripalimab→ SCRT→ CAPOX + Toripalimab:65	130	NA	NA	NA	NA	2021/5-2023/12	(60)
LARC	NCT05176964	2	Neoadjuvant	SCRT→ CAPOX + Tislelizumab	50	NA	NA	NA	NA	2021/12-2024/12	(61)
LARC (Stage II-III)	NCT03854799	2	Neoadjuvant	CRT with Avelumab→ TME	MSI-H:1, MSS:38, Unkown: 62	(22, 23)	(59, 61.5)	(12, 12)	NA	2019/8-2023/12	(62)
dMMR/pMMR CC	NCT03026140	2	Neoadjuvant	Ipilimumab→ Nivolumab	dMMR:32 pMMR:30	dMMR: (22, 68.8) pMMR: (3, 10)	dMMR: (31, 96.9) pMMR: (7, 23.3)	(7, 12)	(35, 100)	2017/3-2024/12	(35)
dMMR/MSI-H CC	NCT03026140	2	Neoadjuvant	Ipilimumab→ Nivolumab	112	(72, 67)	(102, 95)	(5, 4)	NA	2017/3-2024/12	(37)
LARC	NCT05420584	2	Neoadjuvant	chemotherapy→ Tislelizumab	30	NA	NA	NA	NA	2022/11-2024/12	(63)
dMMR/MSI-H CRC	NCT03926338	2	Neoadjuvant	Toripalimab: 17 Toripalimab + Celecoxib: 17	34	(15, 88) (11, 65)	NA	(1, 3)	(34, 100)	2019/5-2024/5	(17)
dMMR RC	NCT04165772	2	Neoadjuvant	dostarlimab→ CRT	16	(12, 100)	NA	0	NA	2019/12-2025/11	(64)
dMMR/pMMR CRC	NA	NA	Neoadjuvant	Tremelimumab + Durvalumab→ Durvalumab	pMMR:21 dMMR:2	dMMR: (2, 100) pMMR: (2, 9)	dMMR: (2, 100) pMMR: (5, 22)	(5, 22)	(17, 74)	2016/11-2019/11	(36)
LARC	NCT02921256	2	Neoadjuvant	PA: FOLFOX + NCRT + Pembrolizumab: 90 CA: FOLFOX +: 95	185	PA: (22, 31.9) CA: (20, 29.4)	NA	PA: (33, 48.2) CA:(25, 37.3)	PA: (65, 94) CA: (61, 89.4)	2016/10-2023/3	(65)
MSS/MSI-H LARC	NCT04231552	2	Neoadjuvant	SCRT→ CapeOX + Camrelizumab→ Surgery	dMMR: 1 pMMR: 28 Unknown: 1	dMMR: (1, 100) pMMR: (12, 46.2)	NA	(8, 26.7)	(27, 100)	2019/11-2022/9	(50)
LARC	NA	3	Neoadjuvant	SCRT→ chemotherapy + Camrelizumab:37 SCRT→ chemotherapy: 61	980	(18, 49.2) (13, 21.6)	NA	(26, 26.6)	(1, 1.6) (0, 0)	2015/1-2021/12	(66)
LARC (Stage II-III)	NCT04083365	2	Neoadjuvant	CRT +Durvalumab→ Surgery	60	(19, 34.5)	NA	(4, 7.3)	NA	2019/11-2021/8	(67)
LARC	NCT03503630	2	Neoadjuvant	SCRT→FOLFOX6 + Avelumab→ TME	44	(15, 37.5)	(27, 67.5)	(31, 70.5)	NA	2018/7-2024/6	(68)
dMMR LARC	NCT04340401	2	Neoadjuvant	CapeOX + Camrelizumab→ Radiotherapy→ CapeOX→ Surgery	27	(7, 33.3)	(7, 33.3)	Lymphopenia: 24 Diarrhea: 8 Thrombocytopenia:4	NA	2020/5-2022/8	(69)
CRC	NA	NA	Neoadjuvant	PD-1:26dMMR CapeOx + PD-1 + SCRT/LCRT: 68pMMR	94	dMMR: (15, 57.7) pMMR: (24, 35.3)	(17, 65.4) (40, 58.8)	(35, 37.2)	(94, 100)	2017/1-2021/10	(70)
LARC	NA	NA	Neoadjuvant	PD-1 inhibitors or cytotoxic chemotherapy	73	(22, 59.5)	(23, 62.2)	(8, 11.0)	(38, 100)	2017/10-2021/12	(71)
dMMR/MSI⁃H LACRC	NA	NA	Neoadjuvant	Sintilimab	11	(10, 90.9)	(11, 100)	0	NA	2020/6-2022/6	(72)
dMMR LARC	NCT04304209	2	Neoadjuvant	Sintilimab	17	pCR:(3, 17.6) cCR: (9, 52.9)	(12, 75)	(1, 16)	NA	2016/10-2022/6	(73)
MSS/MSI-H LARC	NCT02948348	1/2	perioperative	CRT + Nivolumab→ surgery→ FOLFOX or XELOX	MSS:37, MSI-H5	MSS: (11, 30) MSI-H: (3, 60)	NA	MSS: (4, 10.3) MSH:0	NA	2017/1-2020/12	(74)
dMMR/MSI⁃H CRCs	NA	NA	perioperative	neoadjuvant PD-1 inhibitor: 32 perioperative PD-1 inhibitor: 22	32	(22, 75.9)	NA	0	(29, 100)	2019/6-2021/6	(75)

NA, not available; CRC, colorectal cancer; CC, colon cancer; CRT, chemoradiotherapy; pCR, pathologic complete response; MPR, major pathological response rate; cCR, clinical complete remission; TEAEs, Treatment-emergent Adverse Events; OS, Overall Survival; LARC, locally advanced rectal cancer; MSI-H/dMMR, high microsatellite instability/deficient mismatch repair; pMMR, mismatch repair-proficient; MSS, microsatellite stable; TME, total mesorectal excision.

Therefore, neoadjuvant monotherapy based on ICIs can significantly improve the pCR rate and avoid unnecessary surgeries, especially for those are ineligible for chemotherapy, which may translate into long-term survival benefits for dMMR LACRC, with acceptable safety and a low recurrence rate (64, 76). However, there is still a large proportion of patients who failed to achieve pCR after surgery. Effective adjuvant therapy may be necessary to reduce micro-diseases and prevent recurrence and metastasis (57). While there are limited researches on adjuvant ICIs monotherapy. Lynch syndrome is a common form of familial CRC associated with alterations in four DNA MMR genes (80). A case report shows that a Lynch syndrome patient with peritoneal metastasis received nivolumab as adjuvant therapy, achieving pCR, and no recurrence was observed during a 9-month follow-up (81). A retrospective study (75) suggests that dMMR/MSI⁃H LACRC patients who have received neoadjuvant immunotherapy can further improve their pCR rate to 75.9% by combining adjuvant anti-PD-1 treatment based on their postoperative efficacy. These researches indicated that adjuvant ICIs monotherapy can be a promising option for mCRC and LACRC. To further determine this advantage, a phase III clinical trial (NCT03803553) will evaluate the efficacy of adjuvant PD-I inhibitor (nivolumab) versus standard adjuvant chemotherapy in MSI-H CRC patients (75).

### 3.2 Immunotherapy doublet therapy

#### 3.2.1 Immunotherapy combinations

In the NICHE study (NCT03026140) (35), early-stage dMMR colon cancer patients receiving neoadjuvant CTLA-4 inhibitor (ipilimumab) and PD-1inhibitor (nivolumab) gained better pathological responses (5). The NICHE 2 study further expanded the sample size, with a pCR rate of 67% (72/112) and 5 patients experiencing 3/4 grade AEs (37). However, this combination did not indicate significant improvement in pMMR patients (35). Similarly, compared to perioperative chemotherapy (82), combining anti-CTLA-4 (tremelimumab) and anti-PD-L1 (durvalumab) did not significantly prolong median relapse-free survival (9.7 months) and overall survival (24.5 months) in pMMR CRC patients with liver metastasis (36). Numerous studies have confirmed the efficacy and safety of immunotherapy combinations, which has promoted the NCCN guidelines (v2.2022) to recommend nivolumab ± ipilimumab or pembrolizumab as neoadjuvant treatment options for resectable dMMR/MSI-H mCRC (35, 83). But current immunotherapy combinations did not improve the efficacy of pMMR patients with early-stage CRC or mCRC significantly. Nonetheless, the safety of immunotherapy combinations has been confirmed for pMMR CRC (36, 83).

#### 3.2.2 Chemotherapy and immunotherapy combination

Recently, two studies on immunotherapy combined with chemotherapy are underway, which will address the issue of whether the synergistic effect can also appear in perioperative therapy of LACRC. ATOMIC study (NCT02912559) (84) is exploring the efficacy of PD-L1 inhibitors (atezolizumab) combined with chemotherapy versus adjuvant chemotherapy in dMMR stage III CRC, with the primary endpoint being disease-free survival. Although the POLE EDM indicates a better response of CRC to immunotherapy plus chemotherapy, there seems to be no similar improvement in advanced CRC (43, 85). Therefore, the POLEM trial (NCT03827044) (85) aims to investigate whether adjuvant chemotherapy combined with PD-L1 inhibitor (avelumab) can improve disease-free survival in stage III dMMR/MSI-H/POLE EDM colon cancer patients.

#### 3.2.3 Radiotherapy and immunotherapy combination

The potential synergistic effect of immunotherapy and radiotherapy has prompted extensive research to validate its efficacy in various cancers (86–89). Not limited to dMMR-mCRC, it was reported that local radiotherapy combined with PD-1 inhibitor (sintilimab) (90) or PD-L1 inhibitor (tislelizumab) (91) can overcome the immune resistance of pMMR mCRC. Several researches are ongoing to investigate whether the synergistic effect appears in LARC. Li et al. are conducting a multicenter Ib phase study to investigate the safety and efficacy of PD-1 inhibitor (sintilimab) combined with radiotherapy for MSI-H/dMMR rectal cancer (92). Another Phase II study will evaluate whether neoadjuvant anti-PD-1therapy (pembrolizumab) and radiotherapy can improve the safety and efficacy of LARC patients (93).

#### 3.2.4 Chemoradiotherapy and immunotherapy combination

Although two researches applying PD-1 inhibitors (nivolumab (74) and pembrolizumab (65), respectively) combined with chemotherapy showed no significant improvement in pCR rate, another study on 980 LARC patients suggested that the pCR rate of the SCRT with immunotherapy (PD-1 inhibitor camrelizumab) group was higher than that of the non-immunotherapy group (49.2% *vs* 21.6%) (66). The significant differences in this large-scale study manifested the effectiveness and safety of combined immunotherapy. In a phase II trial (NCT04231552) (50), patients with advanced rectal cancer received CAPOX combined with PD-1 inhibitor (camrelizumab) after SCRT and reached a higher pCR rate of 46.2% than that of the combination of PD-L1 inhibitor (avelumab) and mFOLFOX6 after SCRT (37.5%) (68). While it is worth noting that the mid-term results of a phase II trial (NCT04911517) (39) showed that in pMMR LARC patients, the combination of LCRT and PD-1 inhibitor (tislelizumab) also achieved a high pCR rate (50.0%). Therefore, despite the shorter radiotherapy time, SCRT may achieve similar efficacy as LCRT, combined with immunotherapy.

Considering that LCRT may increase toxicity and reduce tolerance of patients compared with SCRT, it remains necessary to determine the optimal combination of LARC neoadjuvant therapy. TORCH (NCT04518280) (60) explored the combination of SCRT and PD-1 inhibitor (toripalimab) for the neoadjuvant therapy of LARC. And the preliminary efficacy showed that the pCR rate and CR rate were as high as 56.2% (18/32) and 58.1% (36/62), respectively. This result suggests that SCRT combined with immunotherapy may be more advantageous. Moreover, the REGINA study (NCT04503694) (94) will investigate the efficacy of the combination of PD-1 inhibitor (Nivolumab) and chemotherapy with SCRT, while the PRIME-PR study (NCT04621370) (95) will directly compare the differences in efficacy between LCRT and SCRT in neoadjuvant immunotherapy combined with TNT.

Currently, neoadjuvant chemoradiotherapy followed by total mesorectal excision is considered the optimal treatment for LARC (74). However, recent results have shown that compared to neoadjuvant chemoradiotherapy alone (10.5%-38.0%), long-term radiation therapy (LCRT) or short-term radiation therapy (SCRT) combined with immunotherapy can significantly improve the pCR rate (37.5%-50.0%) of LARC patients without increasing the risk of AEs (96, 97). Moreover, the determination of radiotherapy strategies may further improve the safety of combination therapy.

#### 3.2.5 Targeted therapy and immunotherapy combination

Since cyclooxygenase-2 may mediate immune escape and inflammatory response (98), applying cyclooxygenase-2 inhibitors may elevate the responsiveness of cancer cells to ICIs. To further demonstrate its synergistic effect, a phase II trial (NCT03926338) (17) discovered that PD-1 inhibitor (toripalimab) (99) combined with cyclooxygenase-2 inhibitors (celecoxib) can achieve a higher pCR rate in dMMR/MSI-H LACRC, compared with anti-PD-1 monotherapy (15 (88%) *vs* 11 (65%)). Only 1 case (3%) of grade 3/4 treatment-related AEs was observed. However, in the NICHE study (NCT03026140) (35), pMMR CRC patients who received neoadjuvant CTLA-4 inhibitor (ipilimumab) combined with PD-1 inhibitor (nivolumab) and celecoxib showed no significant improvement. Therefore, the synergistic effect of targeted therapy and immunotherapy seems to be more evident in dMMR/MSI-H LACRC, while the improvement in the efficacy of pMMR CRC is limited.

## 4 Conclusion

Recent studies have shown that compared with chemotherapy or chemoradiotherapy, perioperative immunotherapy-based therapies can significantly improve the pCR rate of dMMR/MSI-H CRC, without increasing AEs or postoperative complications. Meanwhile, the combination strategies are also expected to further improve the efficacy of pMMR patients, especially immunotherapy combined with chemoradiotherapy or radiotherapy. However, recent studies on immunotherapy combinations and the combination of targeted treatment and ICIs seem to have failed to achieve better results in pMMR/MSS CRC. Many promising immunotherapy-based therapies still require expanding sample size and follow-up results. Furthermore, mature biomarkers for identifying CRC patients with therapeutic responses are required. Additionally, limited toxicity of immunotherapy may be related to low doses and shorter treatment duration, while the higher pCR rate may be associated with more treatment cycles and longer treatment intervals (17, 35, 42). Therefore, further research is expected to determine the optimal therapeutic combination, treatment cycle, and dosage for different populations to coordinate the relationship between efficacy and safety (100). In addition, there are many ongoing but not yet reported studies on perioperative immunotherapy for CRC, as shown in **Supplementary Table 1**.

## Author contributions

J-TC: Conceptualization, Investigation, Resources, Supervision, Writing – original draft, Writing – review & editing. Y-WZ: Conceptualization, Funding acquisition, Investigation, Project administration, Supervision, Writing – review & editing. T-RH: Investigation, Resources, Writing – review & editing. J-LW: Investigation, Writing – review & editing. MQ: Conceptualization, Funding acquisition, Methodology, Project administration, Resources, Supervision, Writing – review & editing.
